# Microhomology-Mediated End-Joining Chronicles: Tracing the Evolutionary Footprints of Genome Protection

**DOI:** 10.1146/annurev-cellbio-111822-014426

**Published:** 2024-09-21

**Authors:** Agnel Sfeir, Marcel Tijsterman, Mitch McVey

**Affiliations:** 1 Molecular Biology Program, Sloan Kettering Institute, Memorial Sloan Kettering Cancer Center, New York, NY, USA; 2 Department of Human Genetics, Leiden University Medical Center; Institute of Biology Leiden, Leiden University, Leiden, The Netherlands; 3 Department of Biology, Tufts University, Medford, Massachusetts, USA

**Keywords:** MMEJ, Polθ, gene editing, replication stress, mitotic repair, genetic diversity

## Abstract

The fidelity of genetic information is essential for cellular function and viability. DNA double-strand breaks (DSBs) pose a significant threat to genome integrity, necessitating efficient repair mechanisms. While the predominant repair strategies are usually accurate, paradoxically, error-prone pathways also exist. This review explores recent advances and our understanding of microhomology-mediated end joining (MMEJ), an intrinsically mutagenic DSB repair pathway conserved across organisms. Central to MMEJ is the activity of DNA polymerase theta (Polθ), a specialized polymerase that fuels MMEJ mutagenicity. We examine the molecular intricacies underlying MMEJ activity and discuss its function during mitosis, where the activity of Polθ emerges as a last-ditch effort to resolve persistent DSBs, especially when homologous recombination is compromised. We explore the promising therapeutic applications of targeting Polθ in cancer treatment and genome editing. Lastly, we discuss the evolutionary consequences of MMEJ, highlighting its delicate balance between protecting genome integrity and driving genomic diversity.

## INTRODUCTION: THE REPERTOIRE OF DNA DOUBLE-STRAND BREAK REPAIR PATHWAYS

**Figure 1:**
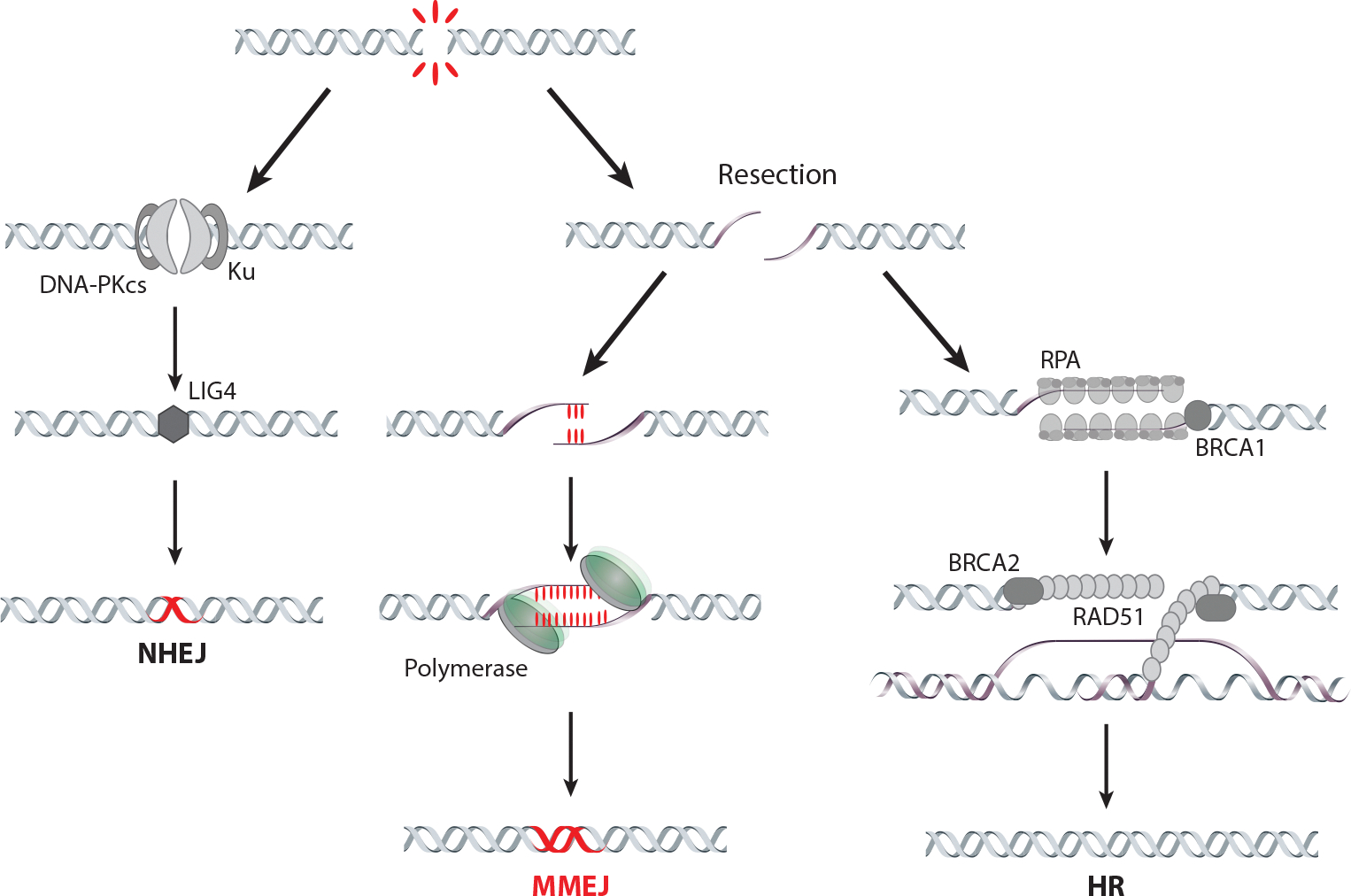
DNA double-strand break repair pathways. NHEJ is a mechanism that directly joins broken DNA ends by involving LIG4, XRCC4, and XLF. During S and G_2_ phases of the cell cycle, DNA end resection and formation of the 3′ single-stranded DNA overhangs are orchestrated by CtBP-interacting protein and the MRE11-RAD50-NBS1 complex. This impedes the NHEJ repair pathway while playing a crucial role in MMEJ and HR. HR, requiring RPA, RAD51, BRCA1, and BRCA2, entails the recognition of complementary sequences in the unbroken sister chromatid or homologous chromosome. DNA base pairing promotes the invasion of the resected end into the homologous DNA duplex and subsequent DNA synthesis utilizing the sister chromatid or homologous chromosome as a template. This review centers on MMEJ, a mechanistically distinct repair pathway with high mutagenic potential. MMEJ repair junctions frequently exhibit deletions and insertions attributed to the activity of low-fidelity polymerases, including Polθ in metazoans. Abbreviations: HR, homologous recombination; LIG4, DNA ligase IV; MMEJ, microhomology-mediated end joining; NHEJ, nonhomologous end joining; Polθ, polymerase theta; RPA, replication protein A. Figure adapted from Sfeir & Symington (2015).

DNA damage can have severe consequences for the stability and integrity of the genome. Among the different types of DNA breaks, double-strand breaks (DSBs) are particularly detrimental as they involve the complete severing of both strands of the DNA double helix and can lead to chromosomal translocation and rearrangements. DSBs can arise from various endogenous and exogenous sources. Endogenous triggers include replication errors, spontaneous DNA damage caused by reactive oxygen species, and enzymatic processes such as DNA rearrangements during V(D)J recombination, class switch recombination (CSR), and meiosis. Exogenous insults, including ionizing radiation, chemotherapeutic agents, and environmental mutagens, can directly induce DNA strand breaks or cause other DNA damage, such as bulky adducts or crosslinks, leading to DSBs if not properly repaired (Mehta & Haber 2014, Tubbs & Nussenzweig 2017). To counteract the deleterious effects of DSBs, cells have evolved highly conserved repair mechanisms, primarily categorized into three major pathways: nonhomologous end joining (NHEJ), homologous recombination (HR), and microhomology-mediated end joining (MMEJ) (Figure 1).

NHEJ is active throughout all cell cycle stages and involves the direct ligation of broken DNA ends, enabling fast repair, albeit with the potential for introducing errors. The Ku protein complex, composed of Ku70 and Ku80 subunits, recognizes DSBs and recruits DNA-PKcs, which phosphorylates downstream targets and facilitates DNA end processing. The Artemis protein trims and modifies DNA ends, while the DNA ligase IV-XRCC4 complex ligates the DNA ends together (Chiruvella et al. 2013). HR is an error-free repair pathway that primarily operates during the S and G_2_ phases of the cell cycle, often taking advantage of an intact homologous DNA sequence (Ranjha et al. 2018, San Filippo et al. 2008). During HR, the DNA break undergoes extensive end resection, which results in the generation of single-stranded DNA (ssDNA) overhangs (Daley et al. 2015, Symington 2016) that invade the homologous sequence. This invasion forms a displacement loop structure, where the invading ssDNA is aligned with the complementary sequence on the sister chromatid or homologous chromosomes. The resolution of this intermediate structure can lead to crossover, where genetic material is exchanged between chromatids, or noncrossover, where the invading ssDNA is displaced without exchange. HR requires several key factors to ensure successful repair, including RAD51, which binds to ssDNA overhangs and forms a nucleoprotein filament that mediates the search for homologous sequences. Additionally, BRCA1 assists in DNA end resection, while BRCA2 and PALB2 promote the assembly and stabilization of RAD51 on the ssDNA.

## A HISTORICAL OVERVIEW OF MICROHOMOLOGY-MEDIATED END JOINING ACROSS SPECIES

The mechanism of MMEJ was first proposed in 1986 to explain how breaks with partially complementary 3′ ends could be aligned and ligated in monkey cells transfected with cut plasmids (Roth & Wilson 1986). A decade later, Boulton & Jackson (1996) reported a similar phenomenon in budding yeast deficient in canonical end-joining factors Ku70/80 and DNA ligase IV. Shortly after, a residual joining activity was detected in hamster cells lacking Ku86 and XRCC4 (Kabotyanski et al. 1998) and was shown to be biochemically driven by PARP1 and DNA ligase III (Audebert et al. 2004; Wang et al. 2005, 2006). Initially labeled alternative end joining (A-EJ) owing to its identification in NHEJ-deficient cells, MMEJ occurs even when classical NHEJ repair is available. The spectrum of organisms where MMEJ has been observed is diverse, from *Escherichia coli* (Chayot et al. 2010) to multicellular eukaryotes. MMEJ repair activity has been reported in fission yeast (Almeida & Godinho Ferreira 2013, Decottignies 2007), *Aspergillus fumigatus* (Zhang et al. 2016), *Caenorhabditis elegans* (Roerink et al. 2014, van Schendel et al. 2015), *Drosophila melanogaster* (Khodaverdian et al. 2017, Yu & McVey 2010), *Arabidopsis thaliana* (Gallego et al. 2003, van Attikum et al. 2003), *Trypanosoma brucei* (Glover et al. 2011), *Danio rerio* (He et al. 2015), *Mus musculus* (Yousefzadeh & Wood 2013), and *Homo sapiens* (Bennardo et al. 2008, Deng et al. 2018, Truong et al. 2013).

A-EJ was initially observed when HR and NHEJ were compromised (Ceccaldi et al. 2015, Mateos-Gomez et al. 2015, Wyatt et al. 2016), leading to its association as a backup end-joining pathway. However, subsequent research indicated a more prominent role for this pathway, operating even when HR and NHEJ are present. A characteristic feature of A-EJ across different species is the presence of microhomologous sequences of varying lengths at repair junction sites. This signature led to the descriptive term of MMEJ (Ma et al. 2003). MMEJ in metazoans heavily relies on DNA polymerase theta (Polθ), thus giving rise to the term theta-mediated end joining (TMEJ) (Schimmel et al. 2019). Given the variations in microhomology requirements, the source of insertions, and genetic backgrounds, questions arose regarding whether all these mechanisms are distinct end-joining pathways. However, we propose that A-EJ, TMEJ, and MMEJ fundamentally constitute a dynamic network of interconnected strategies rather than a set of isolated pathways. This adaptability may involve utilizing local sequence features or the presence of available enzymes to enhance the repair process. To avoid pigeonholing these pathways solely to organisms where Polθ is expressed and to acknowledge that the term alternative may no longer be appropriate, in this review, we collectively define all these mechanisms as MMEJ.

**Figure 2:**
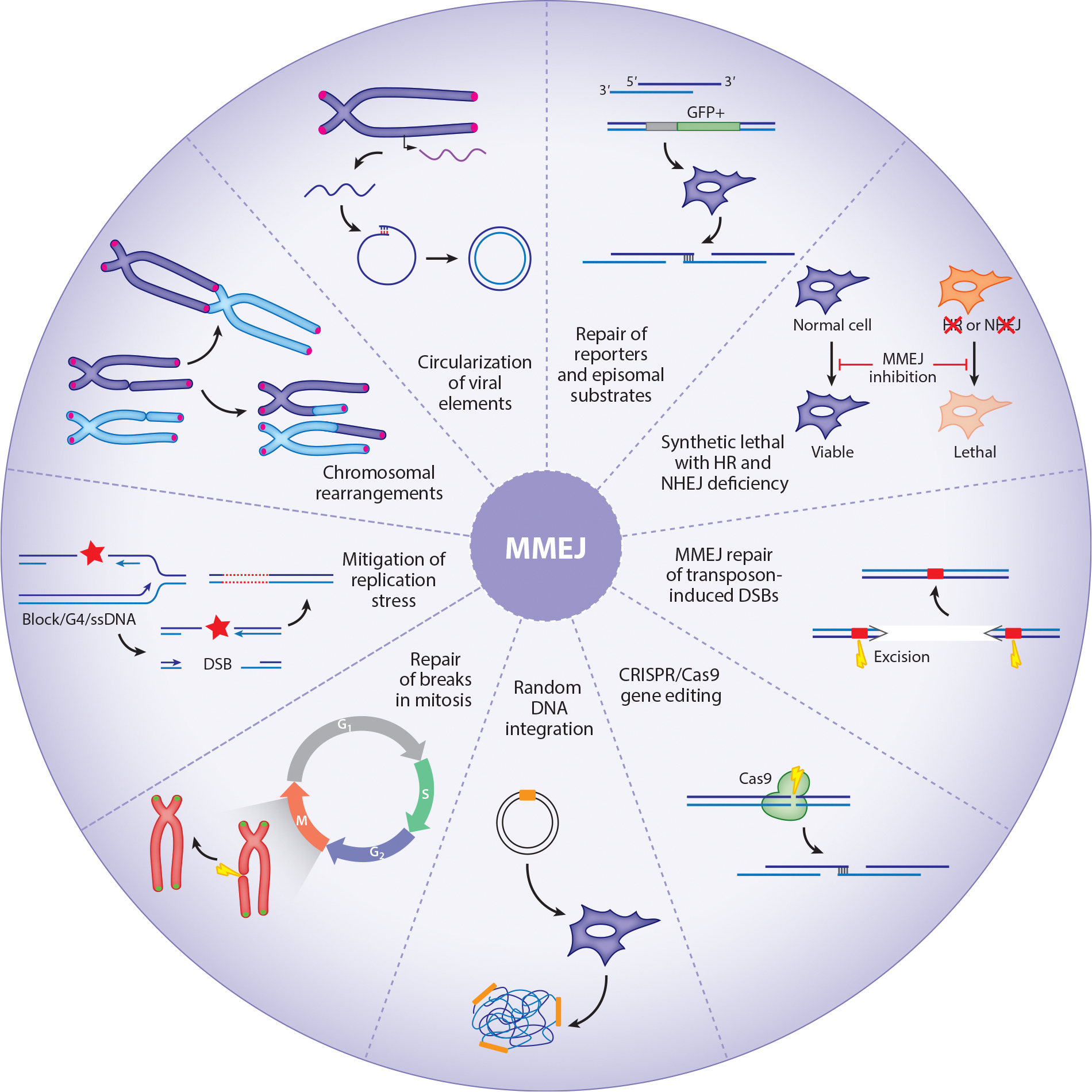
An overview of the activities carried out by MMEJ. MMEJ has been extensively studied using fluorescent reporters and episomal substrates. Its role in DSB repair has also been documented in the context of transposon-induced breaks in *Caenorhabditis elegans* and *Drosophila melanogaster* and during chromosome rearrangements (translocation and telomere fusion) in mouse cells. The activity of MMEJ has also been associated with repairing broken replication forks, a role that is likely carried out during mitosis. Recent investigations have established connections between MMEJ and gene editing facilitated by CRISPR/Cas9 and its involvement in enabling the random integration of DNA into the genome. Lastly, an intriguing and largely unexplored facet of MMEJ has surfaced in its contribution to the circularization of endogenous retroviruses. This multifaceted functionality underscores the significance of MMEJ in various cellular processes. Abbreviations: DSB, double-strand break; HR, homologous recombination; MMEJ, microhomology-mediated end joining; NHEJ, nonhomologous end joining; ssDNA, single-stranded DNA. Figure adapted from Brambati et al. (2020).

MMEJ has been associated with increasing DSB repair-related phenomena, including genome editing, chromosomal rearrangements, mitotic repair, circularization of viral elements, and repair of transposon-induced breaks (Figure 2). In addition, it is important to mitigate replication stress, and its inhibition triggers synthetic lethality in HR-deficient cells. Below, we delve into the multifaceted roles that MMEJ assumes within cells as a guardian against genomic instability and a catalyst for promoting genetic diversity.

## MECHANISMS OF MICROHOMOLOGY-MEDIATED END JOINING: CONSERVED LOGIC, BUT DIFFERENT PLAYERS

**Figure 3:**
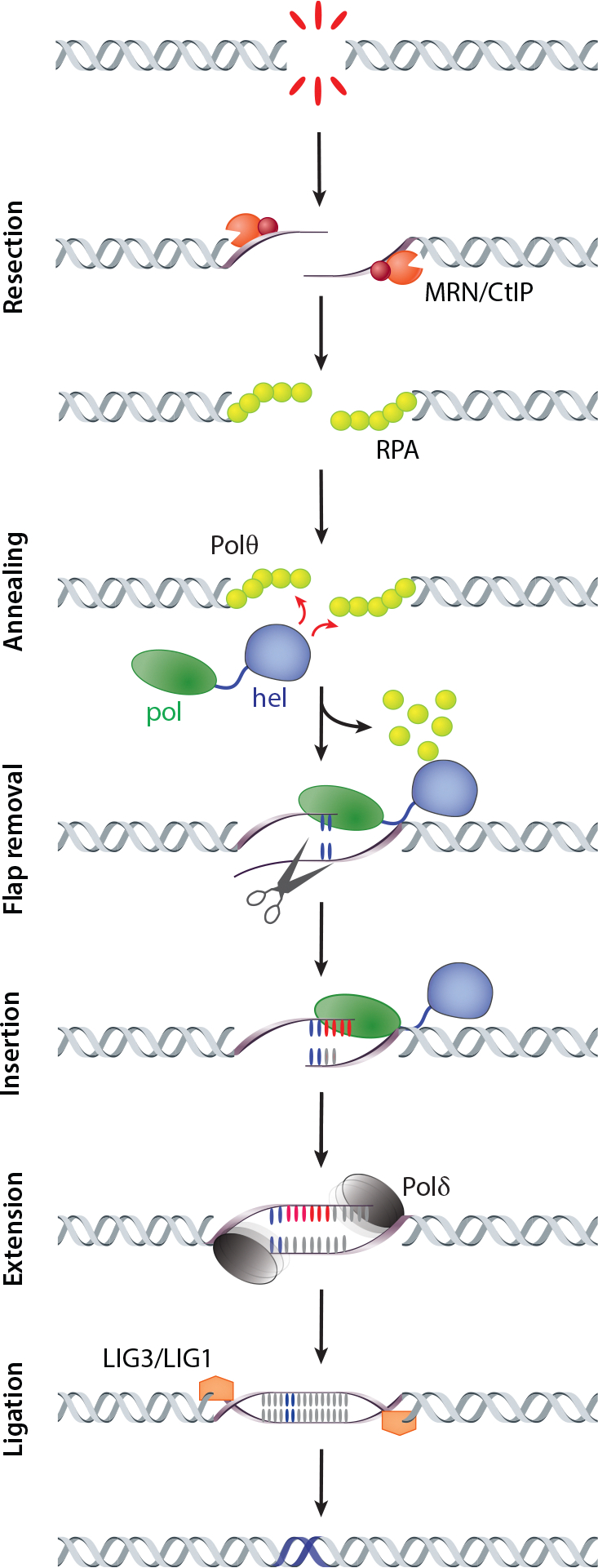
Molecular mechanism of MMEJ. The mechanistic basis of MMEJ, depicted largely in the context of the metazoan pathway, involves several steps, each contributing to the efficient repair of double-strand breaks. The process initiates with DNA end resection, exposing regions of microhomology—short complementary sequences. These exposed microhomologies then serve as sites for the annealing of single-stranded DNA, facilitating the alignment of the broken DNA ends. Following the annealing step, flap removal occurs, removing any nonhomologous overhangs that might be present. Subsequently, the annealed substrate undergoes fill-in synthesis by DNA polymerases. The final step in the MMEJ mechanism is the ligation of nicked DNA, creating a continuous and fully repaired DNA molecule. Abbreviations: hel, helicase; MMEJ, microhomology-mediated end joining; MRN, MRE11-RAD50-NBS1; LIG, ligase; pol, polymerase; Polδ, polymerase delta; Polθ, polymerase theta; RPA, replication protein A. Figure adapted from Sfeir & Symington (2015).

Once MMEJ was established as a bona fide repair mechanism, attention turned to the protein factors required for its execution. At its core, DSB repair by MMEJ requires end resection to produce ssDNA, clearance of bound proteins to allow for synapsis and annealing at microhomologous sequences, removal of nonhomologous 3′ tails, DNA synthesis to fill single-stranded gaps, and ligation (Figure 3). Depending on the nature of the DSB, some of these steps may not be required. Furthermore, the exact details of MMEJ can diverge depending on the organism. For example, the amount of microhomology required for synapsis during MMEJ appears to vary widely, with 1-nt microhomologies prevalent in *C. elegans* (Koole et al. 2014) and 6–20-nt microhomologies frequently utilized in budding yeast (Lee & Lee 2007). Interestingly, in cases where preexisting microhomologies are absent, DNA secondary structures, such as stem-loops, can form and provide intramolecular primer-template junctions for synthesizing nascent microhomologies, which can be used in MMEJ (Khodaverdian et al. 2017, Yu & McVey 2010).

Many of the proteins required for each stage of MMEJ have been identified (Figure 3). Resection occurs via the same protein complexes that are responsible for generating 3′ ssDNA in HR: Mre11-Rad50-Xrs2 (MRX) and Sgs1-Exo1 in yeast and MRE11-RAD50-NBS1 (MRN) and BLM-EXO1 in metazoans (Truong et al. 2013). Synapsis of broken ends is promoted largely through the action of DNA polymerases. In yeast, polymerases lambda (Polλ) and delta (Polδ) cooperate during MMEJ, with Polλ synapsing DNA ends, followed by Polδ synthesis to fill in single-stranded gaps (Meyer et al. 2015, Wilson & Lieber 1999). Polymerase zeta (Polζ) may also be involved in certain contexts (Lee & Lee 2007).

Polθ, possessing both helicase and polymerase domains, plays a central role in MMEJ in metazoans and plants. Initial synapsis is accomplished through the Polθ helicase domain (Newman et al. 2015), which can remove replication protein A (RPA) bound to ssDNA, thereby clearing the way for the annealing of flanking microhomology (Mateos-Gomez et al. 2017). In a mechanism that is not fully understood, the synapsed ends are transferred to the polymerase domain, which synthesizes short stretches of nascent DNA using the annealed microhomologies as primers (Zahn et al. 2015). A recent study provides evidence for a handoff from Polθ to the more processive Polδ, especially in contexts that require filling of larger single-stranded gaps (Stroik et al. 2023). Several subpathways of MMEJ, utilizing different polymerases, have also been postulated. In vitro, Polλ and polymerase beta (Polβ) have been shown to promote MMEJ, with Polβ promoting CAG triplet repeat instability (Crespan et al. 2012). Interestingly, Polλ is also involved in MMEJ in mammalian cells, independent of Polθ (Chandramouly et al. 2023). The mechanisms that promote the preferred usage of different polymerases during MMEJ remain to be characterized.

In budding yeast, the removal of long (6–20-nt) nonhomologous tails before the synthesis step of MMEJ is primarily carried out by the Rad1-Rad10 nuclease (Lee & Lee 2007). A recent study also provided evidence that the proofreading (3′–5′ exonuclease) activity of Polδ might be involved (Shaltz & Jinks-Robertson 2023). At least two metazoan proteins involved in removing 3′ flaps have been identified, including the APE2 nuclease (Fleury et al. 2023) and Polδ exonuclease activity (Stroik et al. 2023). Further study is required to understand the contexts that promote the use of these different pathways.

Following the fill-in synthesis of ssDNA gaps and cleavage of any 3′ flaps generated through displacement synthesis, MMEJ is completed by DNA ligation. In budding yeast, MMEJ is partially dependent on DNA ligase IV-XRCC4 (Ma et al. 2003), while in metazoans, DNA ligase IV is specifically involved in NHEJ. In its place, the DNA ligase III-XRCC1 complex plays a dominant role in the final MMEJ ligation step in zebrafish (He et al. 2015) and mammals (Simsek et al. 2011, Wang et al. 2005), with DNA ligase I acting in support (Paul et al. 2013, Simsek et al. 2011).

In addition to the core machinery described above, several other proteins play important roles in promoting MMEJ in vivo. One of these is poly (ADP ribose) polymerase 1 (PARP-1), whose activity was first described in the process of single-strand break repair. The critical role of PARP-1 in MMEJ was first established using in vitro rejoining assays (Audebert et al. 2004) and later confirmed in multiple in vivo systems, including *Arabidopsis* (Jia et al. 2013) and mammalian cells (Sfeir & de Lange 2012, Sharma et al. 2015, Wang et al. 2006). PARP-1 catalyzes the addition of poly (ADP ribose) to multiple proteins, including itself, and establishes a highly negatively charged environment conducive to an open chromatin structure and required to recruit many MMEJ factors (Audebert et al. 2004, Jia et al. 2013, Sfeir & de Lange 2012, Sharma et al. 2015, Wang et al. 2006).

**Figure 4:**
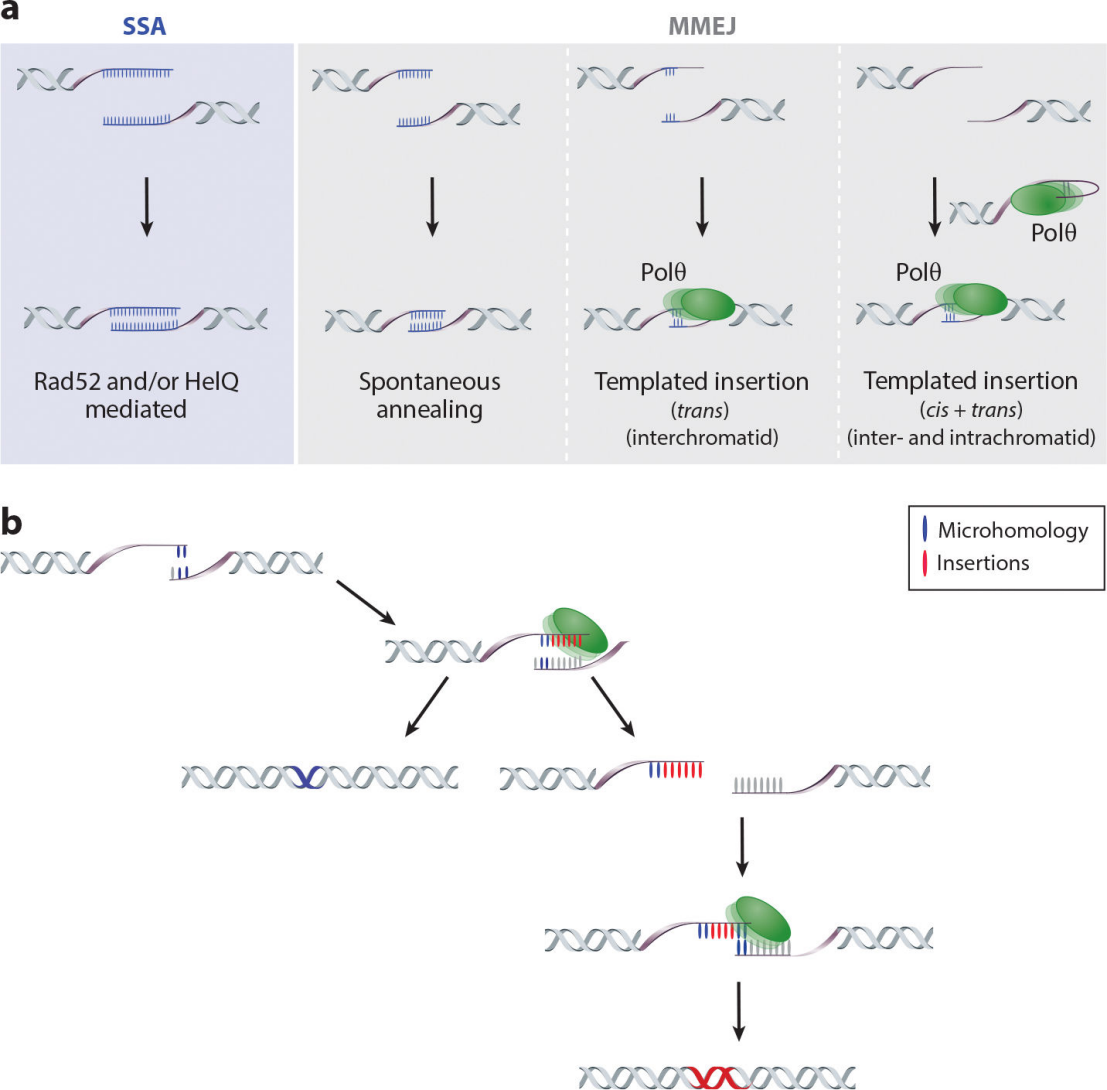
MH and insertion levels across organisms. (*a*) Schematic representation of how the extent of the MH flanking ends of a DSB dictates repair pathway usage and outcome. In a sequence context where extensive direct repeats are present on either side of the DSB, SSA dominates over MMEJ. Smaller direct repeats in close proximity to the DSBs can guide MMEJ, as is seen in budding yeast. Organisms possessing Polθ can repair DSBs through MMEJ with even less of a MH requirement. Polθ extends MHs (≥1 bp) available in the flanks into extended stretches of complementary bases, after which gap filling can commence. The likely template for DNA synthesis is the opposing DSB end, but other mechanisms, including intramolecular priming and synthesis, have also been suggested. (*b*) Schematic illustration to explain the two typical mutational outcomes of MMEJ. Annealing at MHs followed by Polθ-mediated extension results in a deletion hallmarked by MH (*blue*). Dissociation of the substrate before joining, followed by reannealing of the extended end to another microhomologous sequence (*blue*), results in a templated insertion (*red*). Multiple iterations can result in more complex insertions. Abbreviations: DSB, double-strand break; MH, microhomology; MMEJ, microhomology-mediated end joining; Polθ, polymerase theta; SSA, single-strand annealing.

The extent of microhomology required for MMEJ varies across different systems (Figure 4a). As the length of the microhomologous sequence increases, the genetic prerequisites for rejoining deviate from those associated with typical MMEJ. For example, HELQ, which promotes HR, is important for removing RPA from ssDNA and annealing longer microhomologies (Anand et al. 2022, Kamp et al. 2021). Notably, in yeast and mammals, RAD52 becomes significant in the annealing process of long homologies that exceed 20 bp (Fishman-Lobell et al. 1992, Stark et al. 2002, Sugawara et al. 2000), leading to the distinction between MMEJ and single-strand annealing (SSA) (Mehta & Haber 2014, Villarreal et al. 2012) (Figure 4a). However, the difference between MMEJ and SSA is not always clear-cut, suggesting that these pathways may exist along a continuum of repair options contingent upon the specific cellular and genomic context.

## INSERTIONS: KEY FEATURE OF MICROHOMOLOGY-MEDIATED END JOINING BEYOND SIMPLE ANNEALING

In addition to the characteristic microhomology found at deletion junctions, another hallmark of MMEJ in both plants and metazoans is the presence of small insertions generated through DNA synthesis using the flanking sequences around the DSB as a template (Figure 4). A substantial body of work across various genetic systems has firmly established a causal relationship between these templated insertions and Polθ. Early studies in *Drosophila* first reported Polθ-dependent insertions generated upon the repair of I-SceI-induced DSBs (Chan et al. 2010, McVey et al. 2004). Also, in *C. elegans*, Polθ-dependent templated insertions were found at repair sites of DSBs produced by G-quadruplexes, base damage, DNA transposition, or CRISPR/Cas9 (Koole et al. 2014, Roerink et al. 2014, van Schendel et al. 2015). In mouse cells, templated insertions were first found in the DNA joints produced by immunoglobulin CSR: 9% of CSR joints contained insertions of 2–35 bp, and those greater than 10 bp could be mapped to sequences flanking the junction site. This class was absent in B cells lacking Polθ (Yousefzadeh et al. 2014). Subsequently, Polθ was found responsible for nucleotide insertions at unprotected telomeres in mouse embryonic fibroblasts (MEFs) and at translocation junctions in stem cells derived from those MEFs (Kent et al. 2016; Mateos-Gomez et al. 2015, 2017). Also, more recent mutational profiling of repair at CRISPR/Cas9-induced DNA breaks in mouse, fish, plant, and human cell lines revealed templated insertions representing a minor but significant fraction of repair outcomes, which, when tested, were all dependent on Polθ. Together, these studies have provided evidence that templated insertions are an evolutionarily conserved outcome of DSB repair and are a smoking gun for Polθ activity in studies where the role of Polθ cannot be experimentally addressed [e.g., genomes of human populations (Schimmel et al. 2019)].

While the result of Polθ action, the precise mechanism underpinning templated insertions remains unclear. In a simple model, complementary bases flanking a DSB anneal to generate a minimal primer for Polθ. As DNA extension begins at one end, utilizing the other strand as a template, two potential scenarios unfold (Figure 4b). First, there is the possibility of continuation and further processing, ultimately resulting in deletion with microhomology at the junction. Alternatively, there is the chance of dissociation, where the extended end serves as a new substrate for another round of Polθ-mediated extension, culminating in templated insertions (Khodaverdian et al. 2017, van Schendel et al. 2016). A detailed analysis of thousands of templated insertions across various species (e.g., worms, flies, plants, and mammalian cells) supports the idea that the initial steps in the formation of templated insertions and microhomology-mediated deletions are similar; both are predominantly primed by minimal base pairing (Bennardo et al. 2008, Carvajal-Garcia et al. 2020, Hanscom et al. 2022, van Kregten et al. 2016, van Schendel et al. 2016). This observation suggests that the divergence between the two outcomes stems from a distinct downstream step. Despite the frequent occurrence of templated insertions (up to ~35% in *A. thaliana*), what drives discontinuity in MMEJ is still unknown. Intriguingly, the fraction of templated insertions and their iterative character are more pronounced in HELQ mutant *C. elegans* (Kamp et al. 2021), whereas plants do not encode HELQ. This suggests that when MMEJ is disrupted, the default response is to reassemble the dissociated strands on the complementary sequence and proceed with the repair process.

Templated insertions frequently exhibit complex combinatorial configurations reflecting a molecular patchwork composed of multiple smaller segments of different origin (van Kregten et al. 2016). Occasionally, such segments are interspersed with bases whose origin cannot be reliably deduced. These interspersed bases may be templated but simply too small to be mapped accurately. However, they may also reflect Polθ misincorporations or result from translesion synthesis action on damaged nucleotides in the template (Seki & Wood 2008, Seki et al. 2004). Templated insertions are generally small (<25 bp), which is noteworthy, considering that Polθ was shown to be a rather processive enzyme in vitro (Arana et al. 2008). It is conceivable that ssDNA binding proteins that coat resected DSB ends, such as RPA or Rad51, obstruct Polθ activity, thus restricting extensive templated DNA synthesis (Kamp et al. 2021, Mateos-Gomez et al. 2017, Schaub et al. 2022). Nonetheless, templated insertions occasionally encompass >20 consecutive bases, indicating that even thermodynamically stable intermediates can be disrupted before the two opposite DSB ends are irreversibly joined. Whether Polθ dissociates from the template in this process or facilitates template switching through an unknown mechanism is an interesting question, as the latter option could broaden the resolving potential of MMEJ. The plethora of outcome configurations indicates that MMEJ can involve reiterative steps of priming, extension, and dissociation, potentially with species-specific differences.

## MICROHOMOLOGY-MEDIATED END JOINING AS A SAFEGUARD FOR FAILED HOMOLOGOUS RECOMBINATION, MITIGATING REPLICATION STRESS

Considering the relative accuracy of NHEJ on physiological DSBs and the fidelity of HR, questions arise about the necessity and evolutionary selection pressure for MMEJ, a pathway intrinsically characterized by mutagenicity. The observation that numerous de novo rearrangements in mammalian genomes exhibit characteristics of MMEJ (Kloosterman et al. 2015, Lin et al. 2010, Weinstock et al. 2007) suggests that a sufficient number of DSBs are generated during gametogenesis or development that necessitate this specific repair pathway. To provide insight into the number of MMEJ events per cell division, *C. elegans* genomes were whole genome sequenced after 200 animal generations, revealing only a dozen genomic scars attributable to the action of Polθ (van Schendel et al. 2015). This translates to approximately one MMEJ substrate in 200–300 cell divisions in animals grown under nonchallenged conditions. Additionally, plants grown for many generations exhibited a limited number of genomic scars bearing the hallmarks of MMEJ (Ossowski et al. 2010). Given the observation that, in almost all (animal) systems tested (e.g., nematodes, flies, fish, and mice), embryonic development and growth remain largely or completely unperturbed upon *POLQ* disruption, these data argue that the number of DSBs for which HR and NHEJ do not provide a solution is small (van Schendel et al. 2015).

However, in what scenarios do NHEJ and HR fall short? What types of DSBs cannot be repaired by these extensively studied and evolutionarily conserved pathways? One hypothesis posits that DSBs requiring MMEJ are those generated during DNA replication, whereupon HR repair initiates but fails to complete due to sister chromatid damage (Lemmens et al. 2015). Another likely MMEJ scenario may occur when DNA transposition creates DSBs in both sister chromatids, rare cases when both copies of a DNA transposon jump out of newly replicated DNA. While DSBs induced by Tc1 transposition can be repaired by NHEJ in somatic cells of *C. elegans*, in germ cells, the majority are repaired by HR (Ossowski et al. 2010), similar to what was found in *Drosophila* (Engels et al. 1990). Yet, mutagenic repair of transposon-induced DSBs in *C. elegans* germ cells depends on MMEJ (van Schendel et al. 2015).

The finding that MMEJ impairment is synthetically lethal with mutations in genes involved in both HR and NHEJ, along with the recent discovery that MMEJ operates during mitosis (van Vugt & Tijsterman 2023), strongly implies that the pathway can function as a last resort. It prevents genome fragmentation by joining residual breaks that may persist at the final stages of the cell cycle, just before the segregation of duplicated genomes into daughter cells. In such events, the desirability of intact chromosomes may override repair accuracy.

**Figure 5:**
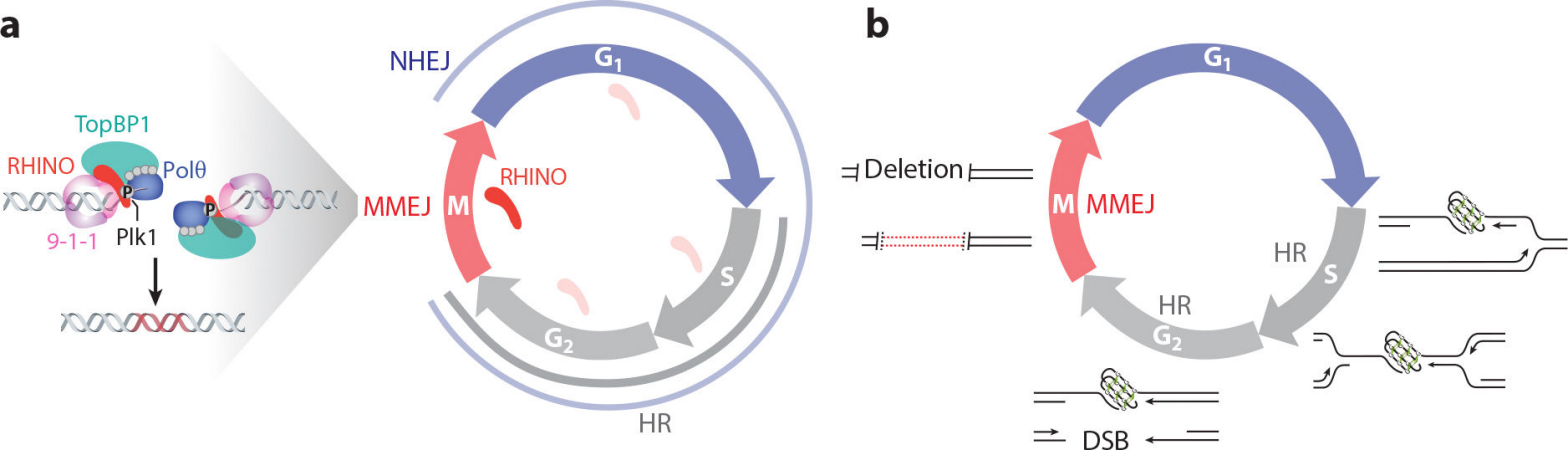
MMEJ activity in mitosis ensures that cells do not commit to cellular divisions with unrepaired breaks originating from the S phase. (*a*) Schematic representation of DNA repair pathways as a function of the cell cycle, with NHEJ and HR being suppressed in the M phase, where only MMEJ is available to fix DNA breaks before cell division. During mitosis, the phosphorylation by PLK1 triggers the assembly of a trimeric complex comprising TopBP1, RHINO, and Polθ. This complex is directed by the 9-1-1 (Rad9-Rad1-Hus1) complex to the DSBs, facilitating the recruitment of Polθ to break sites in the M phase. (*b*) Failure to replicate DNA that contains obstacles, including PARP1 trapped on DNA and G-quadruplexes (depicted in the schematic), creates an ssDNA gap lesion in the nascent strand. This lesion is passed down during mitosis and can potentially cause a DSB in the subsequent S phase. The persistence of the lesion on the sister chromatid acts as a barrier for HR; the DSB persists through the S and G_2_ phases to mitosis, where MMEJ repairs it. Abbreviations: DSB, double-strand break; HR, homologous recombination; MMEJ, microhomology-mediated end joining; NHEJ, nonhomologous end joining; Polθ, polymerase theta; ssDNA, single-stranded DNA.

Replication stress can lead to the formation of DSBs and is associated with increased accumulation of ssDNA gaps, posing threats to genome integrity. Early observations indicated that MMEJ is crucial to maintain genome stability in response to fork collapse, particularly at G-quadruplex-forming DNA loci in *C. elegans* (Koole et al. 2014). The absence of dog-1/FANCJ, which typically facilitates replication through G-quadruplex structures (Castillo Bosch et al. 2014), was associated with MMEJ signatures (small deletion and microhomology) accumulating at genomic G-quadruplex sites. Deletion of Polθ in such scenarios resulted in decreased viability, accompanied by a shift from small MMEJ deletions to larger deletions (thousands of base pairs) in the genomes of surviving worms (Roerink et al. 2014). Sporadic G-quadruplexes are not expected to impede overall genome duplication or block cell cycle progression. However, they pose a potent local barrier to nascent strand synthesis, resulting in a small ssDNA gap that persists as cells undergo mitosis (Kruisselbrink et al. 2008). It has been suggested that replicating the gapped strand in the subsequent S phase results in a DSB. Although HR is expected to fix a DSB in the S phase, the persistence of the G-quadruplex on the sister chromatid, which serves as a template strand, obstructs HR. This creates a substrate ideal for MMEJ repair (Lemmens et al. 2015) (Figure 5). The role of MMEJ in ensuring the stability of collapsed forks is conserved in mammalian cells, including ones exposed to polymerase inhibitors, interstrand cross-linking agents, ultraviolet light, and topoisomerase inhibitors. In addition, a genetic screen for sensitizers of cancer cells to G-quadruplex-stabilizing drugs highlighted Polθ inhibition as a vulnerability (Masud et al. 2021).

Another consequence of replication stress is the accumulation of ssDNA gaps behind the replisome. These gaps become evident in scenarios of increased replication fork speed (Maya-Mendoza et al. 2018). Furthermore, it is postulated that these gaps, ordinarily repaired by HR under normal physiological conditions, significantly increase in cells lacking HR. This accumulation contributes to the increased genome instability and heightened toxicity observed in BRCA-mutated cancer cells treated with PARP inhibitors (PARPi) (Cong et al. 2021). Analysis of replication intermediates using electron microscopy revealed that Polθ is critical in filling ssDNA gaps from stalled Okazaki fragments (Mann et al. 2022). Polθ-deficient cells treated with olaparib or depleted of BRCA1 and BRCA2 displayed increased levels of ssDNA behind the replication fork (Belan et al. 2022, Mann et al. 2022, Schrempf et al. 2022). Sealing of these postreplication gaps has been demonstrated to depend on the polymerase and helicase activities of Polθ, with the latter being involved in removing the RPA that coats gaps. The postreplicative gap-filling activity of Polθ has been proposed to underlie its synthetic lethality with HR deficiency and its synergy with PARPi.

Together, these studies raise intriguing questions regarding whether the role of Polθ in ssDNA gap filling is independent of its function in MMEJ and whether postreplicative gap filling occurs before cells exit the S phase or is perhaps delayed until the G_2_/M phases of the cell cycle. The documented direct association of Polθ with ORC1 is consistent with an S-phase role for Polθ (Fernandez-Vidal et al. 2014). However, the mechanism through which the polymerase is selectively recruited postreplication and how it outcompetes more abundant translesion polymerases are yet to be understood.

## MICROHOMOLOGY-MEDIATED END JOINING IS THE LAST-DITCH RESPONSE TO REPAIRING BREAKS BEFORE CELL DIVISION

Unresolved DNA damage occurring during the S and G_2_ phases of the cell cycle has the potential to persist into mitosis. It is established that NHEJ and HR are suppressed during the M phase (Blackford & Stucki 2020, Esashi et al. 2005, Lee et al. 2014, Orthwein et al. 2014). While upstream signaling by ATM is intact, CDK1- and PLK1-driven phosphorylation of downstream targets, including 53BP1 and BRCA2, suppresses these canonical repair pathways (Esashi et al. 2005, Lee et al. 2014, Orthwein et al. 2014). Recent findings highlighted the role of a tethering complex, composed of MDC1-CIP2A-TOPBP1, in holding together broken DNA ends during mitosis until cells progress to the subsequent G_1_ phase to be fixed by NHEJ (Adam et al. 2021, De Marco Zompit et al. 2022, Leimbacher et al. 2019). Several studies provided hints for the role of Polθ and MMEJ in repairing DNA damage during mitosis. A study using *Xenopus* egg extract revealed that entering mitosis before completing DNA replication results in complex rearrangements driven by Polθ (Deng et al. 2019). Furthermore, when under-replicated DNA is transferred into mitotic human cells, Polθ gets enriched at condensed chromosomes, and this activity leads to the generation of sister chromatid exchanges (Heijink et al. 2022). In flies, Polθ was shown to suppress mitotic crossovers, especially when the Holliday junction resolvases SLX4 and GEN1 were deleted (Carvajal-Garcia et al. 2021). Lastly, it has been noted that Rad52 impedes Polθ activity throughout interphase, which delays MMEJ activity until mitosis, where it repairs replication-born errors (Llorens-Agost et al. 2021).

Direct evidence linking Polθ-mediated MMEJ with mitotic repair emerged by analyzing Polθ dynamics in live cells. This investigation revealed that in cells lacking BRCA2, Polθ foci accumulate in mitosis but not in the S phase (Gelot et al. 2023). Polθ recruitment to break sites was driven by PLK1-dependent phosphorylation, enabling its recruitment to the BRCT domain in TOPBP1 (Figure 5a). Cells expressing a PLK1 phosphorylation mutation in Polθ failed to repair breaks in mitosis, revealing that phosphorylation is necessary to drive its accumulation at break sites (Gelot et al. 2023). Introducing a Polθ phosphomimic was sufficient to foster its interaction with TOPBP1 in interphase. Whether this is sufficient to enhance MMEJ activity remains to be established.

An independent mechanism of Polθ recruitment emerged from CRISPR-based screens that identified subunits of the 9-1-1 complex and their interacting partner RHINO as MMEJ factors (Brambati et al. 2023, Hussmann et al. 2021). While 9-1-1 seems to be expressed throughout all cell cycle stages, RHINO expression accumulates strictly during mitosis, where it interacts with Polθ. Interestingly, RHINO was also phosphorylated by PLK1, a process deemed necessary to prompt its interaction with Polθ, which drives its recruitment to damaged sites in mitosis (Figure 5a). Significantly, the selective inhibition of Polθ activity in mitosis phenocopies the synthetic lethality observed when POLQ is genetically depleted in BRCA2-mutated cells. Moreover, the inhibition of Polθ activity in mitosis, but not in interphase, sensitizes BRCA2-deficient cells to PARPi. These studies establish MMEJ as a primary repair pathway in mammalian mitosis (Brambati et al. 2023, Gelot et al. 2023). However, the underlying mechanism that fosters its activity at highly condensed chromosomes remains elusive. Why are the mitotic chromosomes conducive to MMEJ activity, and how do the RHINO-Polθ and TopBP1-Polθ complexes intersect? Furthermore, the genetic relationship between repair by MMEJ and the tethering complex composed of MDC1-CIP2A-TopBP1 (Blackford & Stucki 2020) or mitotic DNA synthesis remains to be investigated (Bhowmick et al. 2023). Ultimately, it remains to be determined whether MMEJ is specifically dedicated to mitotic DSB repair or if a distinct and RHINO-independent MMEJ activity can operate in interphase.

## MICROHOMOLOGY-MEDIATED END JOINING EXHIBITS SYNTHETIC LETHALITY WITH HOMOLOGOUS RECOMBINATION DEFICIENCY, UNDERSCORING ITS POTENTIAL AS A TARGET FOR CANCER THERAPY

MMEJ plays a central role in the genomic instability that underlies many cancers. Multiple studies have demonstrated that MMEJ can promote genome-destabilizing deletions and chromosome translocations (Mateos-Gomez et al. 2015, Simsek et al. 2011, Wyatt et al. 2016). Perhaps unexpectedly, MMEJ can also prevent gross chromosomal rearrangements. In mice, Myc-IgH translocations increase fourfold in the absence of Polθ (Yousefzadeh et al. 2014), while in *Drosophila*, MMEJ suppresses mitotic crossing over (Carvajal-Garcia et al. 2021) and prevents the formation of even larger deletions at transposon-induced DSBs (Chan et al. 2010). The silencing of MMEJ factors is synthetically lethal in cells possessing mutations that inactivate HR and NHEJ. For example, the nuclease activity of APEX2 is required for viability in BRCA2-deficient cells (Fleury et al. 2023, Mengwasser et al. 2019). Similarly, the knockdown of Polθ in HR-deficient cells, including ovarian, breast, and pancreatic tumor cells, triggers cellular lethality (Mateos-Gomez et al. 2015, Oh et al. 2023), and the loss of both Polθ and the HR protein FANCD2 causes synthetic lethality in mice (Ceccaldi et al. 2015). In a remarkable tour de force, a CRISPR screen published in 2019 identified 140 genes that result in synthetic lethality in cells lacking *POLQ*, most of which caused increased levels of replication-associated DSBs (Feng et al. 2019).

Identifying Polθ as a potential target for HR-defective tumors paved the way for developing small molecule inhibitors for clinical application. Inhibitors targeting the polymerase and helicase domains are in clinical trials as a monotherapy in tumors harboring *BRCA* mutations (Bubenik et al. 2022, Zatreanu et al. 2021, Zhou et al. 2021). Preclinical studies indicate that Polθ inhibitors are strong radiosensitizers, arguing for their use in combination therapies. A key factor driving the heightened interest in Polθ in drug discovery is its lack of expression in normal cells, contrasting with its increased presence in various cancers, especially ones with defective HR (Ceccaldi et al. 2015, Higgins et al. 2010, Kawamura et al. 2004, Lemee et al. 2010). Although the underlying basis behind *POLQ* upregulation in cancer remains unknown, it is notable that the elevated Polθ levels in numerous tumor types, such as lung, gastric, pancreatic, and colorectal cancer, correlate with unfavorable clinical outcomes (Wood & Doublié 2016).

Polθ inhibitors synergize strongly with PARPi (Bubenik et al. 2022, Zatreanu et al. 2021, Zhou et al. 2021). Despite the clinical benefits of PARPi treatment, cancer patients invariably develop resistance to these drugs. Thus, there is an unmet need to understand and target PARPi resistance. A common resistance mechanism in patients with nonsense BRCA1/2 mutations occurs through a reversion mutation in which an indel restores the initial reading frame in the compromised *BRCA* gene and activates HR. This event has been observed in vitro and detected in patients, and MMEJ footprints are enriched at reversions (Bhin et al. 2023, Murciano-Goroff et al. 2022, Pettitt et al. 2020, Tobalina et al. 2021). Accordingly, Polθ inhibition in preventing BRCA reversions and delaying PARPi resistance holds significant clinical promise.

## LEVERAGING MICROHOMOLOGY-MEDIATED END JOINING FOR GENE EDITING, FROM PLANTS TO HUMAN CELLS

The advent of CRISPR/Cas9 technology ushered in a new era in genome editing and engineering, offering unprecedented precision and efficiency in manipulating the genetic material of organisms. Typically, editing approaches entail two steps: first, the introduction of a site-specific DSB and, second, the exploitation of cellular repair machinery to fix the breaks. This process can result in genetic alteration, due to either errors introduced during DSB repair or the inclusion of a repair template. The outcome of genome editing strategies may also be influenced by which DSB pathway dominates during the cell cycle phase when the break is introduced (Hustedt & Durocher 2016). Prereplicative DNA breaks (in the G_1_ phase) are predominantly repaired by NHEJ, leading to small deletions. In contrast, DSBs in postreplicative DNA (in the S/G_2_ phase) can be repaired by HR-based pathways, which can be exploited to guide repair toward a desired outcome. For example, including an ssDNA oligo containing stretches of sequences identical to the target DNA, interspersed with the desired sequence alteration, can facilitate precise changes, ranging from a single base to several kilobases incorporating gene expression cassettes (Gallagher & Haber 2021, Ran et al. 2013).

While template-based homology-directed repair (HDR) is well suited for precise genome engineering, its efficiency is very low, primarily because the more efficient end-joining pathways, particularly NHEJ, tend to dominate (Yeh et al. 2019). Interestingly, MMEJ also appears to outcompete HDR, although the reason for this is currently poorly understood. Recognizing that both NHEJ and MMEJ can interfere with HDR, and that MMEJ can substitute for NHEJ (Schimmel et al. 2017, Wyatt et al. 2016, Yu & McVey 2010), several recent studies have explored pharmacological approaches to inhibit both end-joining pathways, leading to remarkable increases in HDR-mediated repair efficiencies (Schimmel et al. 2023, Wimberger et al. 2023), with some instances reaching up to 50% of all targeted alleles in human cell lines. The enhanced HDR could prove especially valuable in treating monogenic disorders where corrected cells gain a selective advantage over noncorrected cells (e.g., in SCID-X1 and certain patients with Fanconi anemia). The ability to direct the outcome of CRISPR technology more effectively toward a desired outcome and suppress adverse end-joining events will facilitate the development of improved tools for basic research and accelerate the development of safer CRISPR-based therapies for the benefit of human health.

Nonetheless, blocking end-joining pathways to facilitate HDR is not a one-size-fits-all solution for all genome editing scenarios. This is particularly true in nondividing cells or tissues where delivering exogenous DNA is challenging. MMEJ may offer a valuable alternative, particularly when precise insertions or deletions are required. For instance, when a genetic defect results from an out-of-frame-causing deletion or insertion, the gene may be restored by introducing a DSB in the vicinity of the target locus, flanked by appropriate small direct repeats. These repeats can guide MMEJ, leading to a favorable outcome (Grajcarek et al. 2019). However, despite its promise, MMEJ may also pose challenges for genome engineering. Its inherently error-prone nature can result in unintended mutations and genomic rearrangements. As researchers continue to refine and understand the intricacies of MMEJ, its role in genome editing is poised to expand, firmly establishing it as an indispensable tool for pushing the boundaries of genetic manipulation.

Apart from its utility as a genome editing tool, MMEJ may also be useful for genome engineering approaches. Long before humans sought to manipulate cells and species through the transformation of foreign DNA, bacteria had mastered the art of inserting segments of their genetic material into host organisms. The aim was to create a more favorable growth environment for their proliferation. For instance, *Agrobacterium tumefaciens* transforms plants by translocating a small portion of its DNA, known as the transferred DNA (T-DNA), into plant cells. Once inside, T-DNA randomly integrates into the plant’s genome, leading to the subsequent expression of *Agrobacterium* (tumor-inducing) genes and the manifestation of crown gall disease (Tzfira & Citovsky 2006). This agrobacterium-mediated transformation has since become the most widely used method for generating transgenic plants, as it allows for manipulating the bacterium to express any desired gene on its T-DNA.

Recent work has uncovered that T-DNA integration occurs through MMEJ: Plants lacking in Polθ were recalcitrant to efficient transformation with *Agrobacterium*, while the signature of Polθ activity was prevalent at the junctions of T-DNA integration sites (Kralemann et al. 2022, van Kregten et al. 2016). Recognizing that Polθ promoted random T-DNA integration into the plant genome opens intriguing possibilities. On the one hand, it raises the prospect of avoiding random integration, thereby improving the quality and biosafety of plant transgenesis by inhibiting MMEJ. On the other hand, a deeper understanding of the critical factors governing MMEJ efficiency and outcomes could pave the way for efficiently engineering the integration of large DNA segments into plant genomes, potentially circumventing the complexities and lower efficiencies associated with HR reactions. The subsequent discovery that MMEJ also operates to integrate exogenously provided (plasmid) DNA in mammalian cells (Saito et al. 2017, Zelensky et al. 2017) indicates that this utility is not restricted to the agricultural domain.

## THE NONPATHOLOGICAL FUNCTION OF MICROHOMOLOGY-MEDIATED END JOINING AS A DRIVER OF GENETIC DIVERSITY

The preservation of a highly mutagenic repair mechanism throughout evolution suggests an inherent nonpathological function for MMEJ. One such function can be driven by the propensity of mutagenic repair to enhance genetic variation and boost genome evolution. The first hints of a role for MMEJ in promoting genetic diversification emerged by examining genomes of wild *C. elegans* isolates. Notably, spontaneously occurring structural variations displayed signatures consistent with Polθ activity (Roerink et al. 2014). Indeed, whole-genome sequencing results revealed that small indels characteristic of the genome in wild-type worms and typical of wild isolates were replaced by extensive deletions in Polθ-deficient worms, highlighting the influence of MMEJ on these genetic changes. Perhaps the most striking showcase for how MMEJ affects genetic variation is the larvacean *Oikopleura dioica*, a species having the most extreme animal genome. Its unmatched genome compaction coincides with extensive rearrangement of genes, which virtually eliminated all ancestral syntenic associations (Deng et al. 2018). This chordate lacks NHEJ and completely relies on MMEJ for repairing breaks that occur outside the S phase, leading to an astonishing reshuffling of its genome.

Interestingly, MMEJ is highly active in embryonic stem cells and during the early stages of development. Specifically, CRISPR/Cas9 breaks induced in the early stages of developing zebrafish embryos depend on MMEJ for repair (Thyme & Schier 2016). In addition, MMEJ signatures dominate at CRISPR/Cas9-induced breaks in mouse embryonic stem cells (Schimmel et al. 2017). The strongest evidence supporting a role for MMEJ in genomic diversification in humans is based on analysis of whole-genome sequencing archived in the ClinVar and Cosmic databases and published in recent literature. This analysis identified templated insertions reflective of MMEJ at breakpoints of structural variations (Schimmel et al. 2019) and other complex genomic rearrangements, including translocation and chromothripsis (Boeva et al. 2013). In addition to being prevalent in several cancer rearrangements, the fingerprints of MMEJ were also detected in other disease-causing alleles of Lynch syndrome and Cornelia de Lange syndrome (Schimmel et al. 2019). The origin of these mutations recently emerged, stemming from the unexpected MMEJ activity at Spo11-induced meiotic DSBs. Analysis of de novo mutations at meiotic hot spots revealed that one in four sperm and 1 in 12 eggs exhibited distinctive footprints indicative of mutagenic repair. MMEJ was a major repair activity on autosomes (Hinch et al. 2023).

## THE BALANCING ACT OF PROTECTING THE GENOME FROM INVADING DNA

Transposons play a pivotal role in genome evolution by contributing to genetic diversity through their ability to mobilize and insert themselves into different genomic locations, influencing gene regulation and potentially driving evolutionary innovation. Excision of DNA transposons creates a DSB at the donor site. In cases such as the *P* element in *D. melanogaster* (Chan et al. 2010, McVey et al. 2004) and Tc1 in germ cells of *C. elegans*, repair relies on Polθ-mediated MMEJ (van Schendel et al. 2015). Experiments also establish a connection between MMEJ and an endonuclease-independent integration mode, where LINE1 retrotransposons invade mammalian genomes. This mechanism often results in truncated insertions with increased microhomology levels and is more prevalent in cells lacking core NHEJ factors (Morrish et al. 2007, Zingler et al. 2005).

Recent work concluded that retrotransposons also exploit MMEJ during different stages of their cycles for efficient mobilization. Nanopore sequencing of HMS Beagle transposable elements in fly oocytes revealed that only 10% of replicated retrotransposon DNA integrates into the genome, while 90% persists as extrachromosomal circular DNA (eccDNA). Upon activation of the retrotransposon, the encoded reverse transcriptase synthesizes the first-strand DNA. A striking observation revealed that MMEJ factors, including ligase III and Polθ, are critical for second-strand synthesis of the long terminal repeat retrotransposon DNA, which involves circularizing the HMS Beagle genome and generating an eccDNA. eccDNA production is critical for replicating the HMS Beagle and, therefore, essential for incorporating new insertions into the linear host genome (Yang et al. 2023). A similar role for MMEJ was reported during the circularization of the retrotransposon mdg4 (also known as gypsy) that naturally mobilizes in somatic tissues, and intracisternal A particle, a mouse long terminal repeat retrotransposon (Yang et al. 2023).

In addition to managing endogenous viral elements, MMEJ plays a critical role in directly controlling the integration of other viruses and exogenous DNA at random positions in the genome, significantly influencing the functionality of both the integrated and host DNA. Within plants, MMEJ activity regulates the integration of T-DNA. At the same time, in human cells, the depletion of Polθ has been observed to hinder most exogenous DNA integrations, with the remaining ones being impeded through the inhibition of NHEJ (Saito et al. 2017, Zelensky et al. 2017). Furthermore, examination of human papillomavirus (HPV) integration sites in cervical cancer has revealed significant microhomology near integration breakpoints, potentially implicating MMEJ in the fusion of viral and human DNA (Hu et al. 2015). Intriguingly, it has been proposed that HPV-encoded oncoproteins may suppress canonical repair pathways as a means to promote MMEJ (Hu et al. 2023, Leeman et al. 2019). The conserved role of MMEJ in controlling the life cycle of selfish genetic elements and extrachromosomal genomes introduces a novel perspective, illustrating how this repair pathway can shape genomic landscapes of linear chromosomes by regulating the degree of insertion from foreign DNA.

## PERSPECTIVE AND OUTSTANDING QUESTIONS

The multifaceted functionality of MMEJ and its widespread conservation across diverse organisms raise intriguing questions about its evolutionary trajectory. Its role as a pivotal regulator in mitosis, specifically repairing unresolved breaks from the S phase, suggests an evolution intricately linked to specific DNA lesions. Substrates lacking homology render them inaccessible to HR, yet end-resected and unrepairable by NHEJ. Such breaks may have imposed an evolutionary pressure on low-fidelity repair pathways. A compelling alternative scenario envisions MMEJ as a strategic player in balancing an arms race between host genomes and invading endogenous and exogenous viruses. MMEJ controls the random integration of exogenous DNA and drives the circularization of extrachromosomal DNA. This scenario posits MMEJ as a mechanism to temper the genetic impact of invading entities, contributing to the overall genomic stability while simultaneously controlling the level of genetic evolution. Whether evolving to cope with specific substrates resulting from defective replication or participating in a broader genomic arms race, MMEJ is a complex and integral component of cellular processes critical to ensuring genome stability.

To further explore the intricate landscape of MMEJ activities across species, several intriguing and unresolved questions need to be addressed (see Future Issues). Existing genetic, cellular, and biochemical assays, being end points in nature, lack the precision required to tackle these inquiries. Enhanced tools, such as those enabling single-molecule analysis, rapid acquisition, and advanced imaging, are essential for addressing these questions more effectively.

## FUTURE ISSUES

1. What underlies the synthetic lethality between MMEJ and NHEJ? Are unrepaired G_1_ breaks in the absence of NHEJ carried over to mitosis to be repaired by MMEJ?
2. What promotes the use of MMEJ? Is it kinetically driven, due to competition between MMEJ and HR repair proteins, or actively promoted by specific signaling mechanisms?
3. Is MMEJ activity strictly restricted to the M phase, or is there another dimension to its activity in other stages of the cell cycle?
4. Does limiting MMEJ activity to mitosis constrain its mutational outcome?
5. What determines whether a Polθ-mediated event creates a simple microhomology join or a templated insertion? How does polymerase choice affect this?
